# Managing adherence, exposure, and toxicity in oral anticancer therapies

**DOI:** 10.1186/s40780-025-00437-2

**Published:** 2025-04-07

**Authors:** Kazuo Kobayashi

**Affiliations:** Department of Pharmacy, The Cancer Institute Hospital of JFCR, 3-8-31 Ariake, Koto-Ku, Tokyo, 135-8550 Japan

**Keywords:** Oral anticancer agents, Adherence, Occupational exposure, Hand-foot skin reaction, Hypothyroidism, Pharmacist intervention, Medication safety, Oncology pharmacy, Patient education, Adverse effect management

## Abstract

The management of adherence, exposure risk, and adverse effects in oral anticancer agents (OAAs) is essential for optimizing patient outcomes in oncology pharmacy. This review highlights key efforts to enhance adherence, reduce occupational exposure, and improve adverse effect management in OAA therapy.

(1) Adherence management.

We evaluated adherence to trifluridine/tipiracil hydrochloride (TFTD) in metastatic colorectal cancer (mCRC) patients, revealing an overall adherence rate of 85.0%. Common factors affecting adherence included nausea, vomiting, and cancer-related pain. Pharmacist-led interventions, including antiemetic therapy and patient education, significantly improved compliance.

(2) Exposure risk management.

A study on spill kit usage found that 91.7% of incidents involved nurses, with most spills occurring in hospital wards. Following a medical safety workshop, compliance with personal protective equipment (PPE) protocols improved to 100%. These findings emphasize the need for continuous safety training and enhanced spill management protocols.

(3) Adverse effect management.

We examined regorafenib-induced adverse effects, particularly hand-foot skin reaction (HFSR) and hypothyroidism. HFSR occurred in 81.4% of patients, with severe cases (≥ Grade 2) associated with prolonged survival. Routine thyroid function monitoring was essential, as 42.8% of patients developed thyroid dysfunction, with 5.7% requiring hormone replacement therapy. Early intervention and supportive care strategies improved treatment tolerability.

This review underscores the importance of pharmacist-driven interventions in enhancing adherence, ensuring occupational safety, and managing adverse effects. Continued research and collaboration are essential to optimize OAA-based therapy and improve patient care in oncology pharmacy.

## Introduction

Pharmacists play a crucial role as the final guardians of pharmacotherapy, ensuring safe and effective medication use across various clinical settings. Particularly in oncology, pharmacists contribute significantly to team-based healthcare by providing individualized pharmaceutical care at a high level [1]. Furthermore, collaboration between hospital and community pharmacists, often referred to as “pharmacy-pharmacy collaboration,” has been emphasized to establish a seamless and continuous system of patient care [2]. In recent years, efforts have been made to reinforce such frameworks, ensuring consistent patient support throughout the treatment process [3].

However, challenges remain, including manpower limitations and issues related to efficient information sharing [4]. Additionally, as pharmacists’ clinical responsibilities continue to expand, the importance of pharmacist-led clinical research has been increasingly recognized [5]. Oncology pharmacotherapy involves high-risk medications, yet sufficient evidence regarding adverse event management and drug interactions is still lacking [6]. Consequently, there is an urgent need for proactive research efforts to bridge these knowledge gaps [7].

Through our experiences in managing pharmacotherapy for cancer patients, we have encountered numerous issues that demand resolution. In response, we have focused on (1) management to improve adherence to OAAs, (2) evaluating occupational exposure risks and safety measures, and (3) optimizing the management of OAA-induced adverse effects through pharmaceutical interventions. This review outlines the key findings from these efforts and discusses their implications for enhancing the safety and efficacy of OAA-based therapy.

## Adherence management

**Fig. 1:**
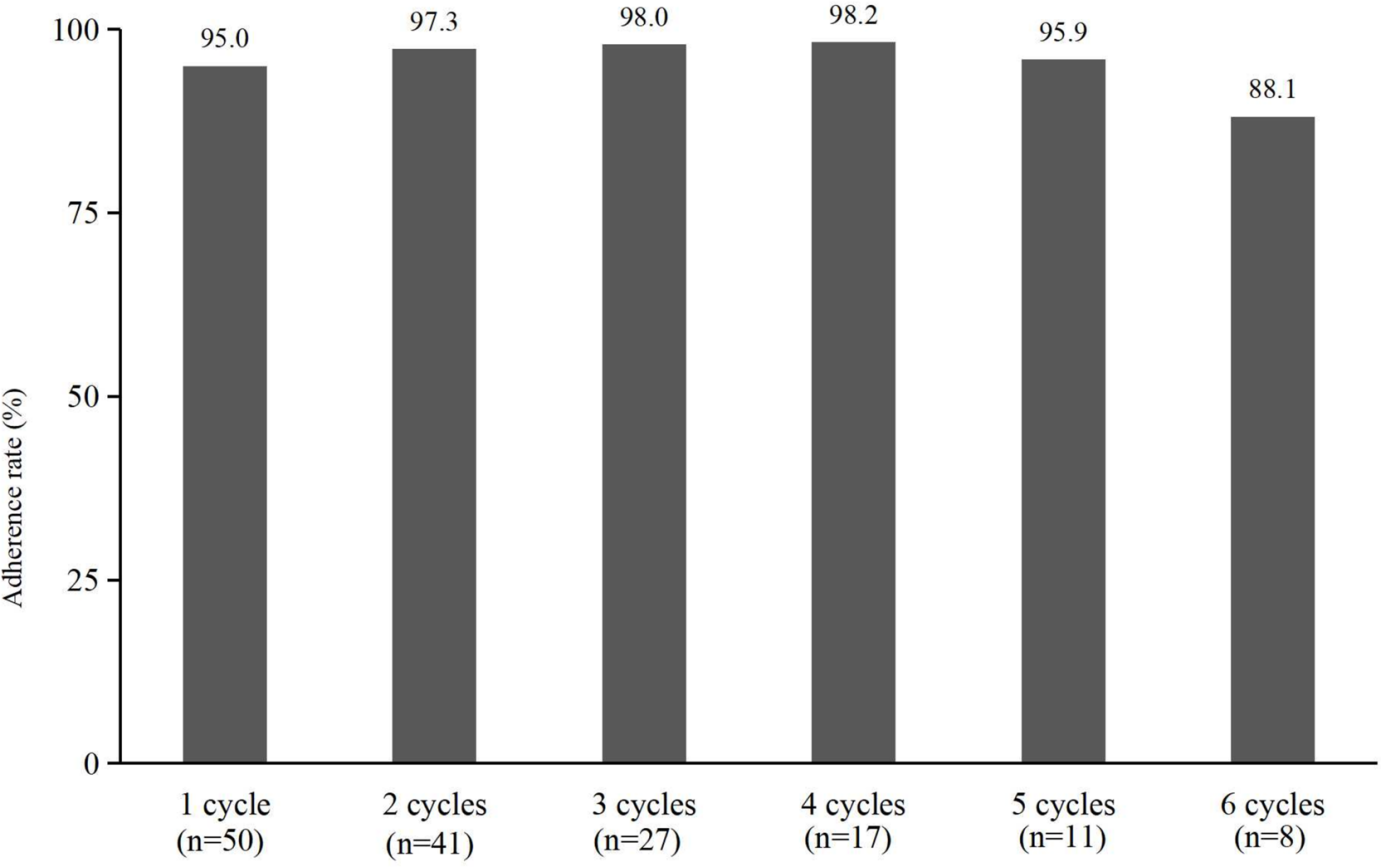
Adherence rate with trifluridine/tipiracil hydrochloride treatment for colorectal cancer

**Fig. 2:**
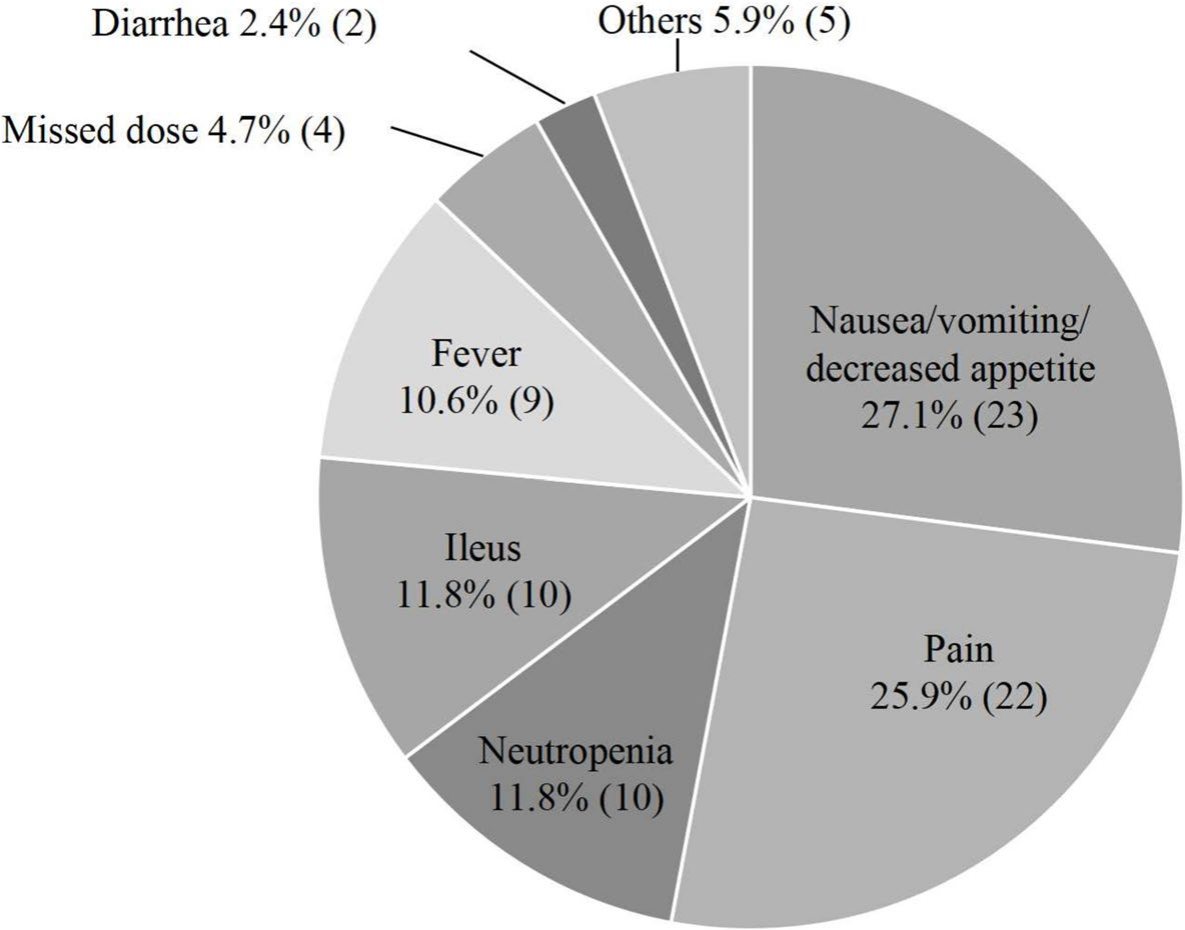
Factors reducing adherence to trifluridine/tipiracil hydrochloride treatment (85 instances)

Adherence to OAAs is essential for achieving optimal therapeutic outcomes, yet it remains a significant challenge in clinical oncology. Given the complexity of dosing regimens and the presence of adverse effects, patient adherence is often compromised, leading to suboptimal efficacy and increased healthcare burdens [8, 9]. We evaluated adherence to trifluridine/tipiracil hydrochloride (TFTD) in metastatic colorectal cancer (mCRC) patients using self-reported adherence studies [10]. A retrospective cohort study examined 50 patients undergoing TFTD treatment and found high adherence rates in the first cycle (95.0%) and subsequent cycles (88.1%–98.2%) (Fig. 1). However, nonadherence was observed in 30% of cases, with major contributing factors including nausea/vomiting (27.1%), pain (25.9%), and neutropenia (11.8%) (Fig. 2). Additionally, patients with poor performance status (ECOG ≥ 1) and those who had received four or more prior regimens exhibited increased nonadherence (Table 1).

**Table 1:** Multivariate model of factors associated with adherence

	Univariate analysis		Multivariate analysis	
Factors	OR (95%CI)	*P*	OR (95%CI)	*P*
Age (< 65 years old)	1.07 (0.28–4.13)	0.58		
Gender (female)	1.21 (0.30–4.86)	0.53		
ECOG performance status (≥ 1)	9.62 (2.11–43.36)	0.01*	25.5 (2.75–236.19)	0.01*
Ccr (≤ 60 mL/min)	3.14 (0.69–14.1)	0.13		
Number of prior regimens (≥ 4)	3.48 (0.86–13.93)	0.07	11.5 (1.24–106.43)	0.03*
Number of metastatic sites (≥ 3)	0.56 (0.10–3.04)	0.40		
Peritoneal metastasis (Yes)	1.71(0.36–8.14)	0.38		
Prior oral chemotherapy (No)	0.68 (0.07–6.51)	0.60		
Opioid treatment (Yes)	3.22 (0.78–13.3)	0.10		
Living status (Living alone)	0.82 (0.70–0.95)	0.15		

*ECOG* Eastern Cooperative Oncology Group, *Ccr* creatinine clearance

*P < 0.05

Our study showed a TFTD adherence rate of over 85.0%, with missed doses at 4.7%, indicating good adherence in most patients. Nausea, vomiting, and appetite loss were the primary factors affecting adherence, followed by cancer-related pain. Effective symptom management, including antiemetic therapy with granisetron, improved adherence. Patients with poor ECOG performance status and multiple prior treatments showed lower adherence in the first cycle. Adherence generally declines with prolonged treatment, requiring continuous patient education and supportive care. In conclusion, side effects and disease progression impact TFTD adherence. Pharmaceutical support and patient education are essential for optimizing adherence and improving treatment outcomes. Future research should focus on refining adherence support strategies, exploring novel digital health solutions, and addressing individual patient needs to ensure sustained adherence to OAAs.

## Exposure risk management

Handling oral anticancer agents (OAAs) presents occupational exposure risks, necessitating appropriate safety measures to protect healthcare professionals. Exposure to anticancer drugs can occur during preparation, administration, patient waste management, and disposal, requiring comprehensive protective measures [11]. Studies have shown that healthcare workers handling cytotoxic drugs may experience contamination through dermal contact, inhalation, and inadvertent ingestion [12, 13].

**Fig. 3:**
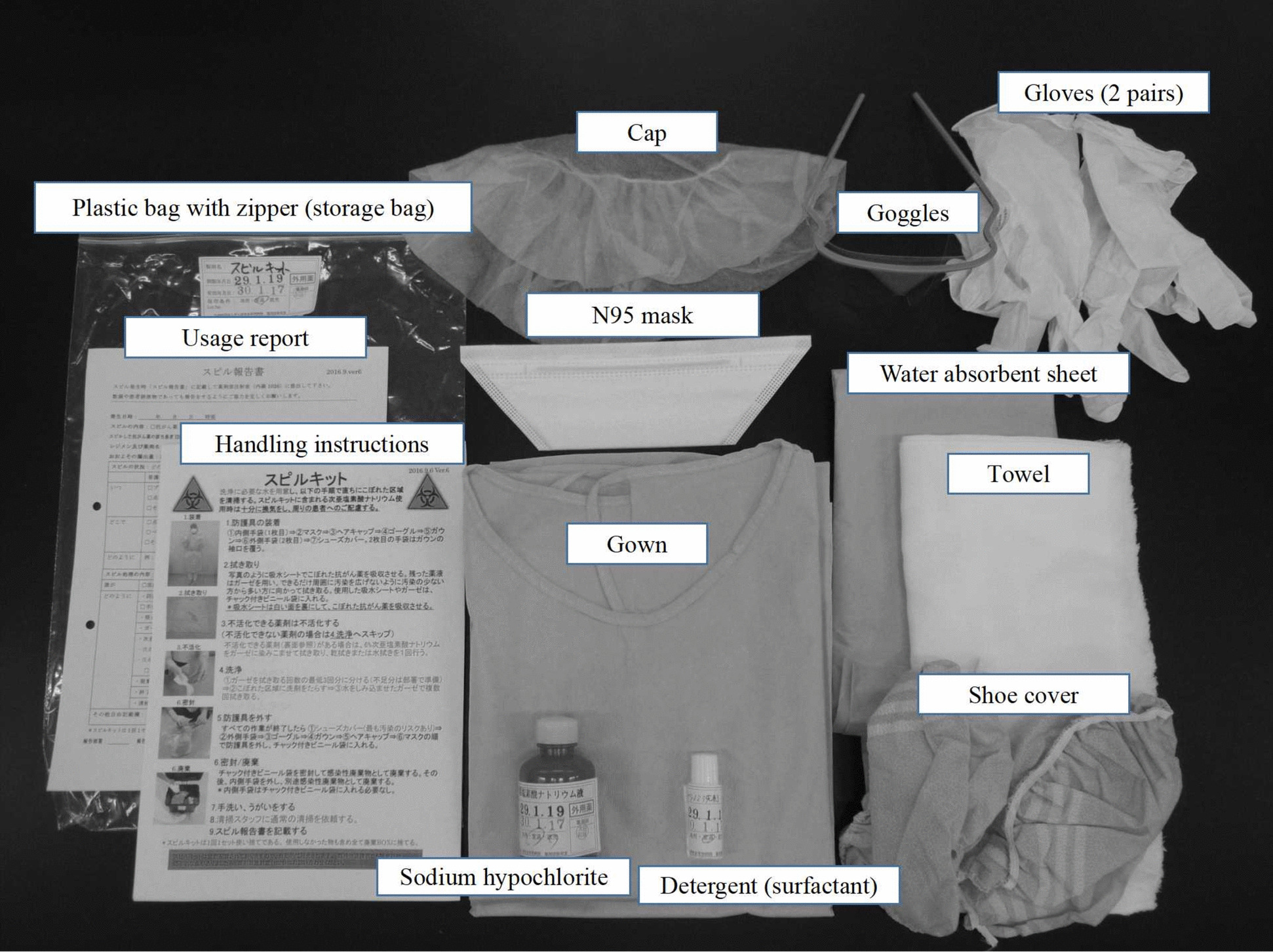
Spill kit configuration

The use of spill kits has been recommended by domestic and international guidelines, including those from the National Institute for Occupational Safety and Health (NIOSH) and the International Society of Oncology Pharmacy Practitioners (ISOPP), to minimize contamination risks [14]. However, reports on their actual use in clinical settings remain limited, and compliance with safety protocols varies across institutions [15]. Further research is needed to assess the effectiveness of spill management strategies and to improve adherence to safety protocols among healthcare professionals. A study investigating spill kit usage from September 2016 to February 2018 revealed 72 reported cases, with 91.7% involving nurses and 79.2% occurring in hospital wards. Spill kits (Fig. 3). were primarily utilized to manage accidental drug leaks during intravenous chemotherapy administration at bedside locations. Notably, compliance with personal protective equipment (PPE) protocols improved significantly following a medical safety workshop, reaching a 100% compliance rate (*P* = 0.026) (Table 2). These findings highlight the importance of continued safety training to ensure proper exposure mitigation practice [16].

**Table 2:** Comparison of Spill Kit Usage Before and After the Medical Safety Training Session (*n* = 72)

	Before the workshop (May 2017—September 2017) *n* = 24	After the workshop (October 2017—February 2018) *n* = 18	*P*-value
Department used			
Hospital wards	19 (79.2%)	15 (83.3%)	
Outpatient	3 (12.5%)	3 (16.7%)	
Pharmaceutical department	2 (8.3%)	0	
Location of use			
Around the bed	13 (54.2%)	11 (61.1%)	
Toilet and washbasin	4 (16.6%)	6 (33.3%)	
Treatment room	4 (16.6%)	1 (5.5%)	
Pharmaceutical department	2 (8.3%)	0	
Corridors and visiting rooms	1 (4.2%)	0	
Spill content			
Anticancer drug	12 (50.0%)	5 (27.7%)	
Urine	7 (29.1%)	7 (38.8%)	
Stool	4 (16.6%)	4 (22.2%)	
Emesma	1 (4.2%)	2 (11.1%)	
Occupation			
Physician	0	1 (5.5%)	
Nurse	22 (91.6%)	17 (94.4%)	
Pharmacist	2 (8.3%)	0	
Years of Occupational Experience			
More than 5 years	9 (37.5%)	9 (50.0%)	
Compliance with personal protective equipment			
Gloves	24 (100%)	18 (100%)	> 0.99
Gown	24 (100%)	18 (100%)	> 0.99
N95 mask	18 (75.0%)	18 (100%)	0.026
Cap	23 (95.8%)	18 (100%)	0.571
Shoe cover	22 (91.6%)	18 (100%)	0.321
Goggles	21 (87.5%)	18 (100%)	0.176
Water absorbent sheet	23 (95.8%)	17 (94.4%)	0.571
Towel	24 (100%)	18 (100%)	> 0.99
Compliance with inactivators and detergents			
Sodium hypochlorite	24 (100%)	24 (100%)	> 0.99
Detergent (surfactant)	24 (100%)	24 (100%)	> 0.99

Further analysis of the spill kit reports indicated that the majority of spills occurred due to infusion system disconnections or leakage at catheter insertion sites. To address these concerns, institutions should consider implementing closed-system drug transfer devices (CSTDs) and reinforced tubing connections. Additionally, routine environmental contamination assessments can help identify high-risk exposure areas and guide preventive interventions.

The importance of spill kit utilization extends beyond healthcare professionals to patients and caregivers managing chemotherapy at home. In outpatient settings, providing simplified spill kits along with comprehensive education on safe handling practices can further reduce exposure risks. Future initiatives should focus on the standardization of exposure prevention protocols across healthcare institutions and the integration of digital reporting systems for real-time spill monitoring.

## Adverse effect management

Oral anticancer agents (OAAs) are associated with a range of adverse effects, necessitating proactive management to optimize therapeutic outcomes and maintain patients' quality of life [17]. Among these adverse effects, hand-foot skin reaction (HFSR) and hypothyroidism are frequently observed in patients receiving regorafenib, a multikinase inhibitor used for metastatic colorectal cancer (mCRC) [18]. HFSR, a dose-limiting toxicity of regorafenib, is characterized by painful erythema and desquamation, particularly on pressure-bearing areas of the hands and feet [19]. Studies have demonstrated that prophylactic skin care measures, including urea-based creams and keratolytic agents, can mitigate the severity of HFSR and improve treatment adherence [20, 21]. Similarly, regorafenib-induced hypothyroidism has been reported as a common yet often underrecognized adverse effect, requiring routine thyroid function monitoring and, when necessary, levothyroxine supplementation to maintain endocrine homeostasis [22].

We investigated the incidence, severity, and management strategies for these adverse effects to enhance patient adherence and improve treatment tolerability. Our findings align with existing literature, suggesting that proactive interventions, including patient education, dermatologic prophylaxis, and endocrine monitoring, can significantly improve patient outcomes and quality of life while on regorafenib therapy.

### Regorafenib-induced hand-foot skin reaction

**Fig. 4:**
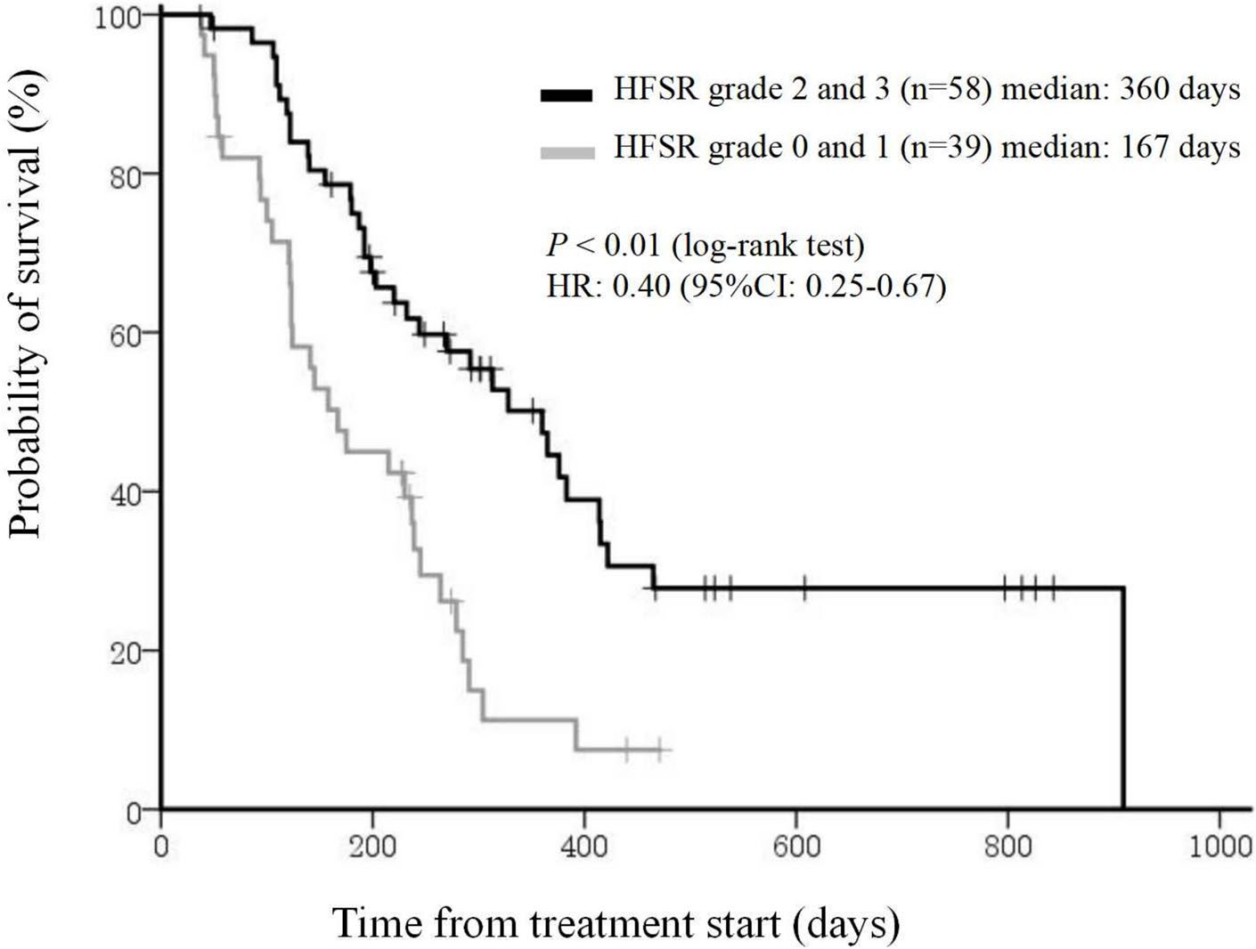
Kaplan–Meier analysis of OS by occurrence of HFSR grade 2 (Yes or No) at any time in regorafenib-treated patients. CI, confidence interval; HFSR, hand-foot skin reaction; HR, hazard ratio; OS, overall survival

**Fig. 5:**
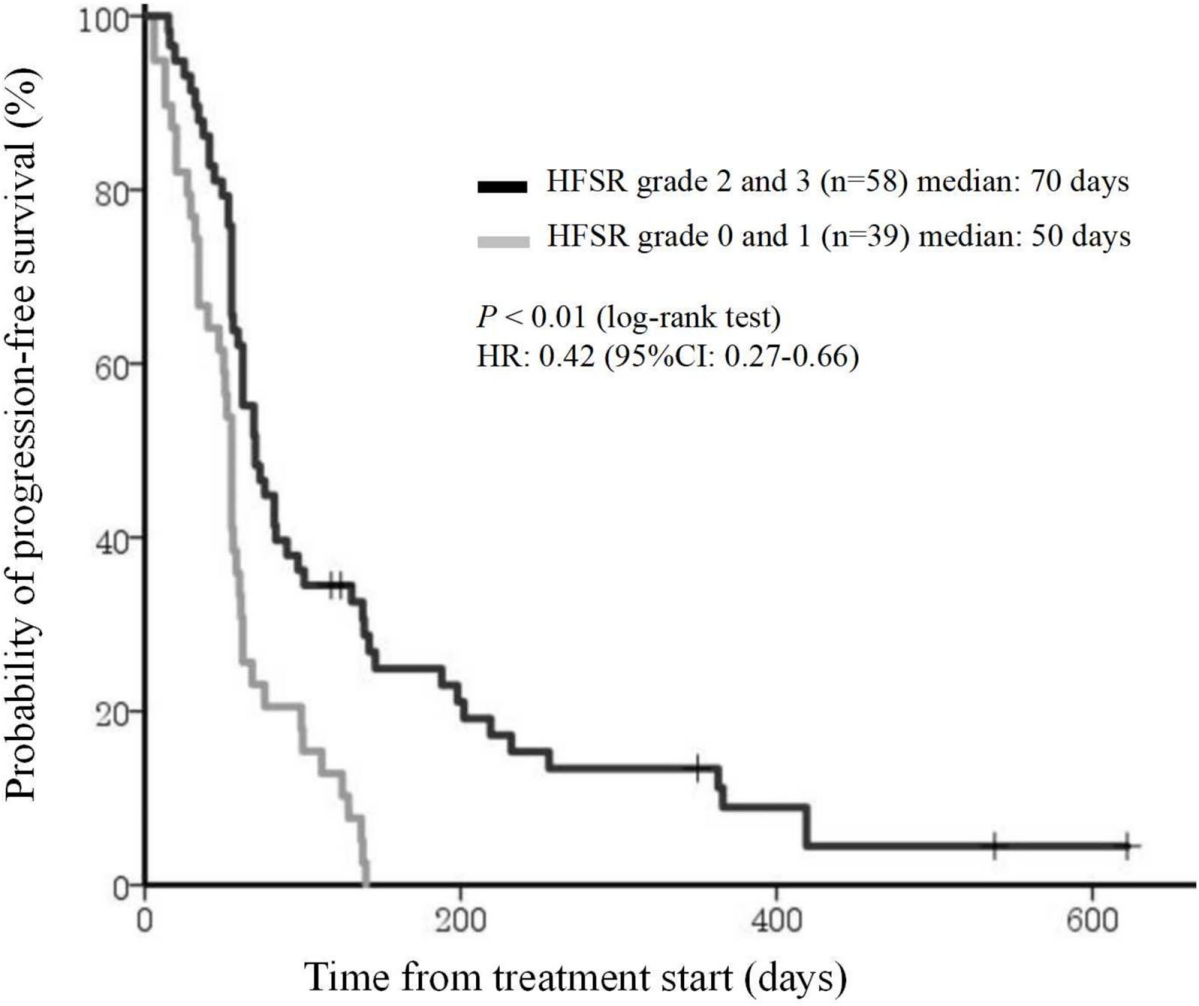
Kaplan–Meier analysis of PFS by occurrence of HFSR grade 2 (Yes or No) at any time in regorafenib-treated patients. CI, confidence interval; HFSR, hand-foot skin reaction; HR, hazard ratio; PFS, progression-free survival

HFSR is a common cutaneous toxicity associated with regorafenib, often leading to treatment discontinuation or dose reduction. Our study examined the incidence and severity of HFSR in patients with mCRC receiving regorafenib and its impact on treatment efficacy. The results revealed that 81.4% of patients experienced HFSR of any grade, with 34.0% developing grade 3 HFSR. Interestingly, patients who developed severe HFSR (grade ≥ 2) exhibited prolonged overall survival (OS) and progression-free survival (PFS) compared to those with mild or no HFSR, suggesting a potential predictive biomarker role for HFSR in regorafenib efficacy [23] (Figs. 4 and 5).

Our study found that 81.4% of patients receiving regorafenib developed hand-foot skin reaction (HFSR), with 34.0% experiencing grade 3 toxicity. This incidence is notably higher than the 47% reported in the CORRECT study, suggesting potential ethnic differences in HFSR susceptibility.

Interestingly, patients with severe HFSR (grade ≥ 2) demonstrated prolonged overall survival (OS) and progression-free survival (PFS) compared to those with mild or no HFSR. This aligns with previous findings, indicating that HFSR may serve as a predictive marker for regorafenib efficacy.The mechanism of HFSR remains unclear, but VEGF inhibition is thought to impair vascular repair in high-pressure areas, leading to inflammation and skin damage. Early onset HFSR, particularly within the first treatment cycle, was associated with improved outcomes, reinforcing its role as a potential biomarker for treatment response.

In clinical practice, proactive management of HFSR through dose adjustments, prophylactic skin care, and early intervention can help maintain treatment adherence while preserving its efficacy. Further research is needed to refine predictive models and individualized management strategies for regorafenib-induced HFSR.

### Regorafenib-induced hypothyroidism

Hypothyroidism is another notable adverse effect of regorafenib, potentially impacting patients' energy levels and overall well-being. Abnormal thyroid-stimulating hormone (TSH) elevation was detected as early as three weeks after treatment initiation, highlighting the need for routine thyroid function monitoring. Hypothyroidism is one of the side-effects caused by regorafenib. In the Japanese subset of the CORRECT study, hypothyroidism developed in 1.5% of the patients, but was not grade 3 or higher in any patient [18]. Symptoms of hypothyroidism include fatigue and dysphonia. Hyperthyroidism must, therefore, be appropriately managed in order to maintain patient quality of life and avoid a critical level of hypothyroidism. In the present study, we clarified the frequency and time of onset of hypothyroidism to improve the management of this side-effect of regorafenib.Our study demonstrated that thyroid dysfunction occurred in 42.8% of patients, with 5.7% developing clinically significant hypothyroidism requiring thyroid hormone replacement therapy [24] (Table 3).

**Table 3:** Thyroid function characteristics of patients treated with regorafenib (*n* = 35)

Number of patients with abnormal TSH elevation (%)	15 (42.8)
Maximal TSH level (mU/L) (%)	
3.68–9.99	11 (31.4)
10.00 or higher	4 (11.4)
Number of patients receiving levothyroxine sodium hydrate (%)	3 (8.5)
Number of patients with abnormal FT4 decrease (%)	2 (5.7)
Number of patients with abnormal FT3 decrease (%)	4 (11.4)
Number of patients with hypothyroidism	2 ( 5.7)
Number of patients with subclinical hypothyroidism	13 (37.1)

Our study found that 42.8% of patients receiving regorafenib experienced thyroid dysfunction, with 5.7% requiring thyroid hormone replacement therapy. This incidence is significantly higher than the 1.5% reported in the CORRECT study, emphasizing the need for careful monitoring.

Thyroid dysfunction was detected as early as three weeks after treatment initiation, suggesting that routine thyroid function tests from the first cycle are essential. The mechanism remains unclear, but VEGFR inhibition may contribute to thyroid capillary regression and ischemic changes, leading to reduced thyroid hormone synthesis.

Clinically, hypothyroidism-related symptoms such as fatigue and cognitive impairment can affect quality of life, making early detection and management crucial. TSH levels should be monitored at the start of each treatment cycle, and thyroid hormone therapy should be initiated when levels exceed 10 mU/L in symptomatic patients.

In summary, regular thyroid function monitoring should be incorporated into regorafenib treatment, with endocrinology consultation considered for severe cases. Further studies are needed to refine optimal management strategies.

## Conclusion

As an oncology pharmacist, I have conducted my clinical practice with the belief that optimizing pharmacotherapy management enhances treatment efficacy and alleviates patient suffering. Through the investigations presented in this review, I have contributed to the establishment of evidence-based clinical practices that can be applied to real-world patient care. Furthermore, this research underscores the significance of pharmacist-led interventions in oncology, demonstrating their essential role in improving adherence, mitigating exposure risks, and managing adverse effects.

Pharmacist-driven evaluations of adverse effect management are directly linked to improving patients’ quality of life. Additionally, the assessment of pharmacotherapy management practices reinforces the importance of pharmacists in the multidisciplinary oncology care team. As clinical pharmacy evolves in response to societal changes, the continuous evaluation of pharmacist-led interventions will be crucial for advancing the profession and expanding its role in patient care.

Moving forward, I am committed to integrating clinical practice and research to further refine pharmacotherapy strategies that optimize patient safety and efficacy. Ongoing efforts to refine adherence management, enhance occupational safety measures, and mitigate adverse effects will contribute to improved cancer treatment outcomes and reinforce the indispensable role of oncology pharmacists.

## Data Availability

No datasets were generated or analysed during the current study.
